# Toward an Effective Innovation Framework for Federal Grant-Making: an Exploration into OPA’s Teen Pregnancy Prevention Program

**DOI:** 10.1007/s11121-023-01582-6

**Published:** 2023-10-10

**Authors:** Mikayla Hyman, Sarah Philbrick

**Affiliations:** Partnership for Public Service, Washington, DC USA

**Keywords:** Grants, Innovation, Adolescent sexual health, Teen pregnancy

## Abstract

States, local, tribal, and territorial governments received almost 20% of all federal spending in fiscal year 2022, about $1.2 trillion dollars (US Office of Management and Budget, 2023a). For a strong, well-functioning democracy, the federal government must be able to disperse grant funds efficiently and effectively. Rates of teen pregnancy in the USA, while decreasing in recent years, are still consistently higher than that of other western industrialized nations (Centers for Disease Control and Prevention, 2023). The Office of Population Affairs’ (OPA) Teen Pregnancy Prevention program combines cutting edge research with innovative grant distribution to creatively tackle this issue. In this commentary, we explore some of the strengths of OPA’s grant distribution program in the hope that others may emulate best practices from this program. Specifically, the special issue highlights how OPA’s grant program took a customer-centered focus by prioritizing work with end users and community. This evidence-based focus enabled the testing of ideas, which in turn could be iterated and scaled up. Through grantee innovation networks, they created space for external expertise, creative thinking, and diversity of thought. Other programs, policymakers, and their partners may learn from OPA’s success, which arises from three core characteristics: (1) their funding stature allows long-term and flexible allocation of funding toward innovation; (2) OPA focuses on creating and supporting local communities and networks of innovation; (3) OPA emphasizes evidence-based decision-making and rigorous evaluation throughout the grant process. With a fifth of the federal budget being dispersed through grant-making and teen pregnancy still at high rates, OPA offers an exciting avenue for innovation and success in both of these areas. This commentary concludes with some recommendations for future practice.

## Introduction

For the past 20 years, the Partnership for Public Service has pursued a mission of building a more effective government that better serves our diverse nation. At the core of this mission is our belief that an innovative, evidence-based, and collaborative government is essential to the long-term prosperity, security, and health of our country. We have worked to realize this vision by supporting and celebrating the best of government—not just ripped-from-the-headlines programs that everyone knows about, but also the critical work done by unheralded civil servants to improve lives. By helping to highlight what works in government and pointing out opportunities for further growth, we believe that we can continue to build a federal organization that is innovative, equitable, and focused on delivering critical services for those who need them most. It is in this spirit of celebration that the Partnership for Public Service is pleased to recognize the innovative work of the Department of Health and Human Services and its grantees to promote new—and rigorously evaluated—ways of supporting adolescent sexual health.

The purpose of this commentary in *Prevention Science* is to identify and define core characteristics of a successful federal innovation program that may be applied in other federal grant programs or prize challenges. This work demonstrates a collaborative approach to public health and program delivery that other programs, policymakers, and their partners may learn from as they seek to increase their impact. The Teen Pregnancy Prevention Program has innovative and effective grant infrastructure and execution which has led to exciting results in adolescent sexual health.

## The Landscape of Grant-making in Government

The federal government employs a number of tools to accomplish policy goals set by Congress, the White House, and others, including the following:

- Direct service delivery (e.g., payments to Social Security beneficiaries or the operation of hospitals by the Department of Veterans Affairs).
- Regulation and enforcement (e.g., the Securities and Exchange Commission’s role in setting and enforcing regulations on financial markets or the FBI’s national security mission).
- Communications (e.g., the Consumer Product Safety Commission’s or Transportation Security Administration’s groundbreaking use of social media to promote safety and security goals).
- Issuing grants to states, localities, other nations, groups, and individuals to perform certain work themselves.

While all of these mechanisms enable our government to better serve the public, grant-making is one of the most effective tools agencies have to scale up their impact as our world continues to grow more complex (Sager, 2018). In fiscal year 2022, the federal government spent in excess of $1.2 trillion in grants, principally in aid to state, local, tribal, and territorial governments (US Office of Management and Budget 2023a). Another $160 billion was spent on research and development and on other programs that invest in the physical, educational, and health infrastructure of the USA (US Office of Management and Budget, 2023b; US Office of Management and Budget, 2023c). These numbers match or exceed what the government spends in other major categories. In 2022, the federal government spent $1.1 trillion on contractors, $701 billion on personnel, and more (USA Spending, 2023). They have also continued to grow historically and may continue to grow in the future as Congress continues to use grants to implement major policy goals (Congressional Research Service, 2019, pp. 33).

Indeed, several large agencies include grant-making and the distribution of federal funds as a core part of their mission. The Small Business Administration, the Federal Emergency Management Agency, and the Economic Development Agency are just a few examples of such agencies. Specifically, the Small Business Administration operates the Small Business Innovation Research and Small Business Technology Transfer programs to encourage small businesses to engage in research and development. Small businesses that gain access to the competitive program can explore their technological potential and are incentivized to profit from its commercialization (Small Business Innovation Research, 2023). The Federal Emergency Management Agency awards grants to “state, local and tribal governments as well as nongovernment entities to help communities prevent, prepare for, protect against, mitigate the effects of, respond to and recover from disasters and terrorist attacks” (Government Accountability Office, 2019, pp. 1). Meanwhile, the Economic Development Agency works with communities to help them build the capacity for economic development based on local business conditions and needs (US Economic Development Administration, 2023).

Many other parts of the federal government also use large grant programs to deliver on their mission. These programs have tremendous impact on the lives of those living in the USA and abroad because they direct funding to areas of critical need, create ecosystems of innovation, invest in underserved communities, and create opportunities for growth. However, some programs have been challenged to manage an uptick in funding as the size and scale of the federal government’s grants footprint have grown. The Government Accountability Office (GAO) has highlighted several ongoing challenges and opportunities involving grants management, including but not limited to further developing the grants workforce, streamlining administrative requirements, clarifying merit-based award criteria, and using evidence to make award decisions (Government Accountability Office, 2017, 2018; Government Accountability Office, 2016a, b).

GAO’s findings and recommendations reflect a profound need to innovate not just the type of work done by grant recipients but also the way the federal government posts, awards, disburses, and manages the grants themselves. Doing so would further unlock the potential of the programs the federal government needs to test new ways of making grants, highlight what works, and reduce administrative burden on both the awarding agency and grantees. To meet these objectives, the federal government should focus on bringing customers and communities into the design process for major grant programs to solicit input, experiment with new ideas, and tailor programs for those they serve.

## A Framework for Customer-focused Innovation

Given the critical role that major grant programs play in the continued health and well-being of the public, government leaders must continue to innovate to stay ahead of evolving threats and opportunities. Based on research and consultation with a wide range of federal innovators, the Partnership for Public Service, in collaboration with Slalom Consulting, developed a framework that highlights 10 key actions used by innovative organizations (Partnership for Public Service, 2019).

First, leadership support can lead to innovation prioritization. When leaders openly and purposefully support innovative efforts, they create the space, motivation, and grace to allow innovation attempts among their employees. Secondly, organizations should empower employees to be creative. Employees who have the freedom and resources to innovate in their day-to-day work can create efficiencies in the short and long terms. Often, innovation can be stifled through outdated infrastructure and processes. It is important for organizations to identify and remove outdated processes, policies, and technologies that prevent innovation.

Organizations can encourage their employees to make small bets on new ideas, iterate, and learn from failure. Innovative organizations encourage experimentation and learning from failures. Organizations can also scale successful initiatives and projects. Organizations should try to establish processes for turning innovative ideas and projects into scalable, sustainable, and effective long-term initiatives when possible. The strategic innovation approach should be specific to the needs of the organization. This may include valuing external expertise, creative thinking, and diversity of thought. Teams should draw on skills from other departments and agencies, academia, and other sectors. Innovative efforts should also be aligned with strategic goals and the mission of the agency. This makes innovation projects more likely to be prioritized, sustained, and valued.

As organizations think about innovation, they should make sure that innovation centers on customer experience, demonstrates strong business practices, and contributes to a larger culture of change. The experience of the customer or the end users is central to an organization’s operations and should be considered paramount to any innovation in an organization. Management practices which are skilled, expert, and standardized create better environments for innovation to flow. Through these characteristics, organizations can build a culture of change where everyone within said organization is willing and able to adapt to change.

While we believe that these characteristics of successful innovation are generally applicable to any government program striving for innovation, they are especially critical for grant-making programs given the size and scale of the positive impact they can have if administered effectively. For grants programs, we believe that three of the actions mentioned above are essential to innovation in program design and delivery.

First, center on the customer experience. Because of the role that grant programs often play in supporting historically underserved communities and the general public, we believe it is essential for these programs to center the customer in their work. The customer may come in many forms—a state Department of Transportation to design safer roadways; a tribe seeking support to relocate in the face of climate change; a community of academics looking for funding to pursue new areas of research; or any other end user or recipient. No matter the focus, it is critical to routinely bring community stakeholders into the design process to provide input, ask questions, test new ideas, and share feedback on what they perceive as working and not working.

Second, make small bets on new ideas, iterate, and learn from failure. Innovative grant programs do not presume to know, at the program officer level, what interventions or solutions will work for a given problem or community. As such, innovative programs may find ways to make small bets on new ideas or unproven programs and gather data to rigorously evaluate their effectiveness. This process may involve issuing planning grants to help recipients mature promising ideas or evaluating new types of programs as a means to encourage innovation.

Third, value external expertise, creative thinking, and diversity of thought. Given the scale of the challenges that some grant programs aim to take on (e.g., new areas of technology, major public health challenges, and more), it is crucial that they find ways to create diverse teams of program officials, awardees, and evaluators who bring different perspectives to the table during design and implementation. When appropriate, these stakeholders should include community groups and practitioners to gather on-the-ground input on what has worked, what has not worked, and what they hypothesize might be effective. These multidisciplinary teams will likely produce solutions that are better tailored to the challenge than those without a diversity of perspectives or thought. Based on our experience, we believe that if programs implement these principles and work collaboratively with communities, they will more effectively serve the diverse needs of the public and forge solutions with demonstrable impact.

## Success of the Teen Pregnancy Prevention Program

The program administered by HHS’ Office of Population Affairs highlighted in this special issue of *Prevention Science* has integrated many of the actions described above into the fabric of its grant initiatives. Alongside the grantees highlighted in this journal, OPA’s work is a testament to the power of using innovation and innovative grant-making to address long-standing public health challenges.

OPA employed each of the major strategies we highlighted in the previous section to great effect over the last several funding cycles. They centered the customer experience. Many grant programs focus on supporting initiatives that have long histories of funding and large bodies of evidence and take conventional approaches to solve challenges. Programs also may involve layers of intermediate governments, grantees, subgrantees, and others between the program office and the end users or communities they are designed to support. By contrast, OPA’s approach to program funding prioritizes different types of engagement with end users and communities to support program outcomes. In particular, the office focuses on providing useful technical assistance and involving as many stakeholders as possible in the design process for the grant program itself. In other words, OPA is distinctly focused on bringing their customers along for the entire journey of a grant cycle.

OPA also makes small bets on new ideas, iterates, and learns from failure. In addition to creating programs that are designed to ideate on new ideas, OPA uses an evidence-based approach that focuses on rigorously evaluating proposed interventions to learn about their efficacy and identify areas for future study as exemplified in *Addressing evaluation barriers with early innovation development for adolescent focused sexual and reproductive health interventions* (Wilson et al., 2023) and *Catalyzing innovation in teen pregnancy prevention: Opportunities, challenges, and lessons learned* (Antonishak et al., 2023). As a result, and after closer analysis, some interventions were identified as promising and effective, while some showed more limited promise. For example, when studying the efficacy of a sexual health video for Black and Hispanic adolescent females, an evaluation team found mixed results in terms of long-term use of contraceptives among the control group, but potentially promising results in individuals’ likelihood to be tested for sexually transmitted diseases—an intervention that is low cost and highly scalable (Jenner et al., 2023). This type of nuanced evaluation—one that aims to understand the efficacy of a program and identify areas for further study—helps weave thoughtful decision-making into every step of the program.

Lastly, OPA values external expertise, creative thinking, and diversity of thought. For each program that received funding, grantees were required to establish an innovation network of stakeholders in a particular community to ideate and test new types of interventions that might improve adolescent sexual health (Office of Population Affairs, 2020). The resulting programs were then run by practitioners and groups working inside that community. For example, Project SHINE: the Sexual Health Innovation Network for Equitable Education was led by a group of experts and self-advocates with intellectual disabilities and included program administrators, care professionals, and others who worked directly with the target community to create new tools for connecting care professionals and youth (Colarossi et al., in press). Embracing diverse perspectives and backgrounds, this type of multidisciplinary program is crucial to unlocking the promise of innovation. Taken together, the Teen Pregnancy Prevention Program is important to celebrate and highlight as an example of a program that has found ways to innovate over time with an unwavering focus on improve health outcomes for America’s youth.

## Lessons Learned for Other Programs

The strategies used by the Teen Pregnancy Prevention Program show the positive difference that government can make when it empowers creative civil servants to focus on mission outcomes, be responsible and creative stewards of the government’s resources, make decisions based on data and evidence, and implement principles of good program design and management. Other programs might consider how they can adopt some of these strategies that have contributed to the success of the Teen Pregnancy Prevention Program.

OPA is empowered to innovate. OPA has been empowered to allocate some of its funding toward innovation in the authorizing statue for the Teen Pregnancy Prevention Program (H.R. 8295, 2022). The statute specially allocates $130,000,000, of which “25 percent shall be available for research and demonstration grants to develop, replicate, refine, and test additional models and innovative strategies for preventing teenage pregnancy” (H.R. 8295, 2022, pp 101). This long-standing allocation is critical to providing the program with the financial flexibility to fund innovative programs and develop new methods to evaluate the success of those programs. Policymakers should consider including similar language as a matter of course in the authorizing statute for similar programs to encourage innovation and give implementing agencies the flexibility to focus on creating innovative outcomes.

OPA focuses on creating and supporting local communities and networks of innovation. At the heart of OPA’s grant programs is the idea that teams based in communities around the country are best positioned to ideate on new solutions that positively influence adolescent sexual health. By specifically setting aside funding for “innovation and impact networks,” the program encourages practitioners to leverage existing relationships and form new ones in pursuit of effective solutions (Ball et al., 2023; Colarossi et al., in press; Hartzler-Weakley et al., 2023). Other programs, particularly those working with historically underserved communities, would do well to learn from this approach as a means to establish durable, knowledgeable programs that drive public impact.

OPA focuses on evidence and evaluation. In addition to an innovation- and community-focused orientation, another core strength of this program is its emphasis on evidence-based decision-making and rigorous evaluation. In particular, OPA’s admission at the outset of its funding that “there is still much to learn about what works, how, for whom and why” and the availability of grant money to evaluate promising solutions emblemizes the office’s focus on evaluation as a core part of the overall program portfolio—not just an afterthought as part of the grant closeout process (Office of Population Affairs, 2020). Other programs might consider elevating the role of evaluation in overall program design to help identify which innovations are worthy of further funding and development.

## Conclusion

In summary, we believe that grant programs like OPA’s Teen Pregnancy Prevention Program represent government at its best: innovative, flexible, community oriented, evidence based, and ultimately focused on delivering for the public. We applaud the good work of the grantees and look forward to continued innovation in this critical public health discipline. At the same time, we encourage policymakers and grant writers to consider more language in authorizing statutes to distribute funds for innovation to build on the impressive work seen at OPA. We also celebrate the civil servants, leaders, technical experts, and others who make this important work possible and look forward to collaborating with our federal partners to highlight best practices like those discussed here so that we may further advance our mission of building a stronger government and a better democracy.
